# Olorin: combining gene flow with exome sequencing in large family studies of complex disease

**DOI:** 10.1093/bioinformatics/bts609

**Published:** 2012-10-10

**Authors:** James A. Morris, Jeffrey C. Barrett

**Affiliations:** Wellcome Trust Sanger Institute, Hinxton, Cambridge, CB10 1HH, UK

## Abstract

**Motivation:** The existence of families with many individuals affected by the same complex disease has long suggested the possibility of rare alleles of high penetrance. In contrast to Mendelian diseases, however, linkage studies have identified very few reproducibly linked loci in diseases such as diabetes and autism. Genome-wide association studies have had greater success with such diseases, but these results explain neither the extreme disease load nor the within-family linkage peaks, of some large pedigrees. Combining linkage information with exome or genome sequencing from large complex disease pedigrees might finally identify family-specific, high-penetrance mutations.

**Results:** Olorin is a tool**,** which integrates gene flow within families with next generation sequencing data to enable the analysis of complex disease pedigrees. Users can interactively filter and prioritize variants based on haplotype sharing across selected individuals **and** other measures of importance, including predicted functional consequence and population frequency.

**Availability:** http://www.sanger.ac.uk/resources/software/olorin

**Contact:** olorin@sanger.ac.uk

## 1 INTRODUCTION

**Fig. 1.**
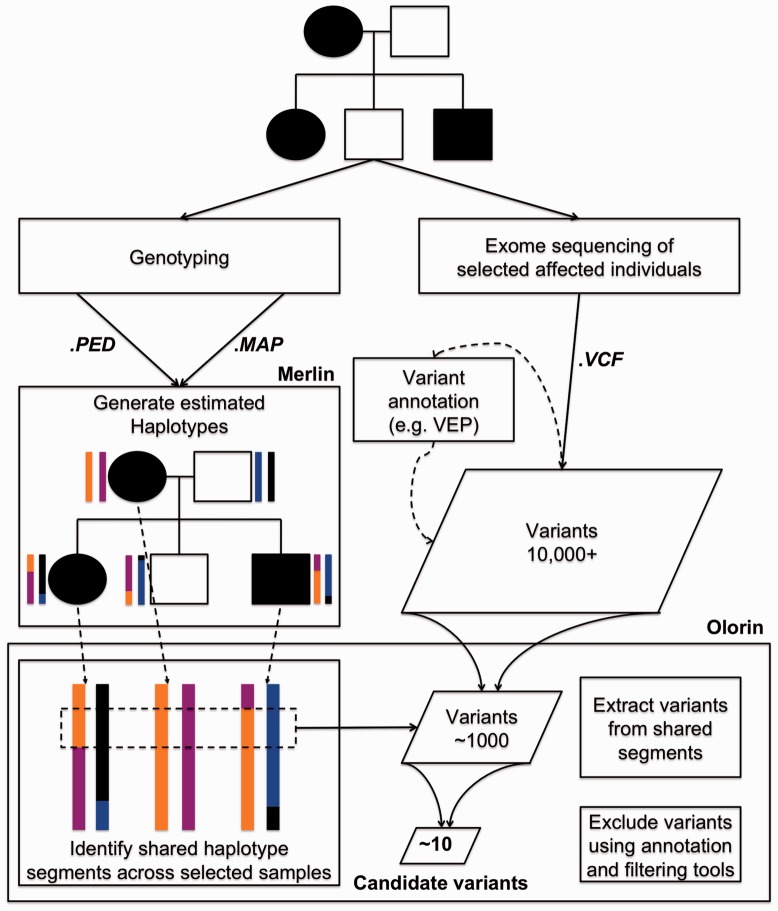
Olorin uses patterns of gene flow estimated by MERLIN to identify genomic regions shared by affected individuals in large pedigrees. This information is combined with next generation sequence data, and only those variants that lie within shared regions are analysed. Users can further refine the list of variants using Olorin’s realtime filtering tools

Next generation sequencing has rapidly become the standard approach for identifying mutations responsible for Mendelian diseases (Bamshad *et al.*, 2011). Although software and file formats for the processing of raw sequence data are relatively robust (Danecek *et al.*, 2011; Li *et al.*, 2009), there is currently a lack of easy-to-use software for downstream analysis of these data. For some study designs, such as focused analysis of fully penetrant *de novo* mutations or autosomal recessive inheritance, exome sequence data can be analysed and filtered relatively simply. Increasingly, however, sequence-based approaches are being applied to complex diseases, which are unlikely to follow a simple genetic model, such as autism (Neale *et al.*, 2012), and to more complicated scenarios, such as large pedigrees with incomplete penetrance. These studies require new tools to enable the diverse community of researchers working on such families to interactively and comprehensively analyze next generation sequence data. Figure 1 shows how our new program, Olorin, integrates within-family linkage analysis with exome sequencing in a user-friendly package.

## 2 FEATURES

### 2.1 File formats

Olorin uses four types of data file: two that provide information about the gene flow calculated by MERLIN (Abecasis *et al.*, 2002), one defining the pedigree structure, and a list of variants identified by sequencing. MERLIN’s haplotyping functionality is used to compute haplotype inheritance within the pedigree. Details of the genomic markers used in the estimation of haplotypes, and pedigree information about the relationships between individuals and their disease status are read from standard.map and.ped MERLIN format files. All variants identified from sequencing across samples need to be provided as a single variant call format (VCF) file (version 4.0 or greater) (Danecek *et al.*, 2011).

### 2.2 Workflow

#### 2.2.1 Selecting individuals

**Fig. 2.**
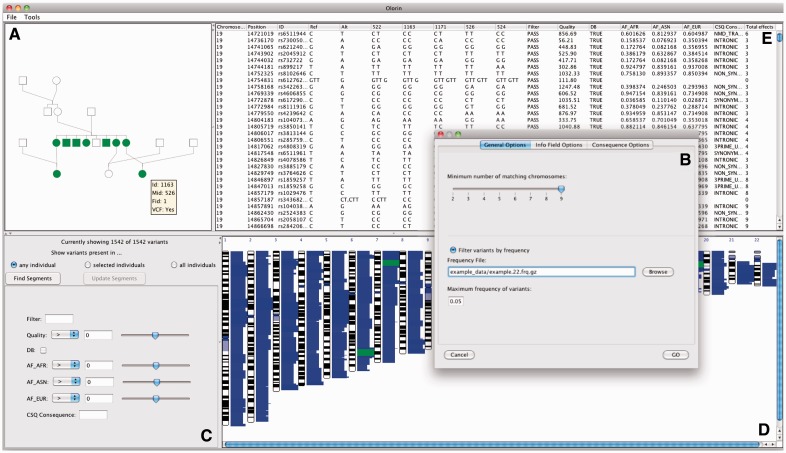
Screenshot of Olorin running on OS X. (**A**) the interactive pedigree panel, (**B**) the general options tab of the filtering dialog, (**C**) the dynamic filtering panel, (**D**) genome-wide segments display, highlighting shared segments in green and (**E**) the variants table

On loading data, Olorin automatically generates an interactive pedigree using standard conventions for information such as sex and disease status. Users can obtain additional information, such as whether a particular individual has been sequenced, via a mouseover popup box. To begin filtering variants, the user first needs to select individuals to be used in searching for shared genomic segments by clicking on them in the pedigree (Fig. 2).

#### 2.2.2 Initial variant filtering

After selecting individuals, the user can customize the analysis via a filtering dialog (Fig. 2). First, they set the minimum number of individuals required to share a segment. This enables searches for variants of incomplete penetrance if the threshold is set below the total number of affected individuals in the pedigree. Next, the user can select which information fields from the VCF will be included for subsequent filtering and display. A population frequency cut-off can also be specified at this point if (as is often the case) the study design is focused on variants expected to be rare in healthy individuals.

#### 2.2.3 Dynamic variant filtering

Olorin populates an analysis table (Fig. 2) with variants found in the shared segments. This table can be sorted on any column, and variants in the table can be filtered out in real time using a number of filtering tools (Fig. 2), which are dynamically generated based on the user-selected data fields. Olorin can show variants discovered in any or all of these individuals, depending on the genetic model under consideration.

#### 2.2.4 Predicted variant effects

Because the ‘consequence’ strings in the VCF information field contain a wealth of parseable information, Olorin supports further processing of two variant consequence string formats: the UK10K analysis pipeline format and the Ensembl Variant Effect Predictor format (McLaren *et al.*, 2010). Because each variant can have multiple consequences, Olorin automatically selects and displays only the most damaging effect for each variant, showing the remainder via a popup box.

## 3 IMPLEMENTATION

Olorin is written in Java and will work on any platform with Java 1.6 or later installed. The interactive pedigree is drawn using the PedVizAPI (Fuchsberger *et al.*, 2008). The genome-wide sharing plots are generated using source code from the visualization tool, IdeogramBrowser (Müller *et al.*, 2007).
